# Individual and Combined Cardioprotective Effects of 1,25(OH)_2_D_3_ and Dimethyl Fumarate in an Isoproterenol‐Induced Myocardial Injury Model

**DOI:** 10.1002/jbt.71081

**Published:** 2026-08-17

**Authors:** Fatma Hande Karpuzoğlu, Serhat Kilinç, İlayda Sözmen, İlknur Bingül, Abdurrahman Fatih Aydin, Aysel Bayram, Semen Önder, Efe Akçe, Semra Doğru‐Abbasoğlu, Müjdat Uysal

**Affiliations:** ^1^ Department of Medical Biochemistry, Istanbul Faculty of Medicine stanbul University, Fatih Istanbul Turkey; ^2^ Department of Pathology, Istanbul Faculty of Medicine Istanbul University, Fatih Istanbul Turkey; ^3^ Istanbul Faculty of Medicine Istanbul University, Fatih Istanbul Turkey

**Keywords:** 1,25(OH)_2_D_3_, acute myocardial infarction (AMI), dimethyl fumarate (DMF), isoproterenol (ISO), NF‐E2‐related factor 2 (Nrf2), NF‐kappa B (NF‐κB), oxidative stress, renin‐angiotensin system (RAS)

## Abstract

Acute myocardial infarction (AMI) is closely associated with excessive oxidative stress, inflammation, and activation of the renin–angiotensin system (RAS). The active form of vitamin D [1,25(OH)_2_D_3_] and the Nrf2 activator dimethyl fumarate (DMF) exhibit antioxidant and anti‐inflammatory effects. This study investigated the individual and combined protective roles of 1,25(OH)_2_D_3_ and DMF in an isoproterenol (ISO)‐induced model of myocardial injury. Male Sprague–Dawley rats received ISO (85 mg/kg, two doses 24 h apart) and were pretreated with 1,25(OH)_2_D_3_ or DMF alone, a combination pretreatment, or a combination post‐treatment. Myocardial injury was assessed using serum biomarkers, histopathology, oxidative stress markers (ROS, MDA, GSH), inflammatory markers (TNF‐α), RAS components (Ang II, AT1R, ACE), and mRNA expression of Nrf2, NQO1, and NF‐κB. ISO administration caused marked myocardial injury, oxidative imbalance, and increased inflammatory and RAS activation while suppressing Nrf2 and NQO1. Both 1,25(OH)_2_D_3_ and DMF significantly reduced oxidative stress, inflammation, and components of the RAS. Combination pretreatment and post‐treatment further improved redox status and molecular markers. These groups showed elevated Nrf2 and NQO1 expression and reduced NF‐κB, AT1R, and ACE expression compared with ISO controls. However, synergistic enhancement was not observed. 1,25(OH)_2_D_3_ and DMF provide both preventive and therapeutic protection against ISO‐induced myocardial injury by modulating oxidative stress, inflammation, the Nrf2 and NF‐κB signaling pathways, and RAS regulation.

## Graphical abstract

1,25(OH)_2_D_3_ and dimethyl fumarate, administered alone or in combination, provide significant cardioprotection against isoproterenol‐induced myocardial injury by reducing oxidative stress, inflammation, and structural damage through modulation of the Nrf2/NF‐κB signaling pathways and the renin–angiotensin system (RAS).

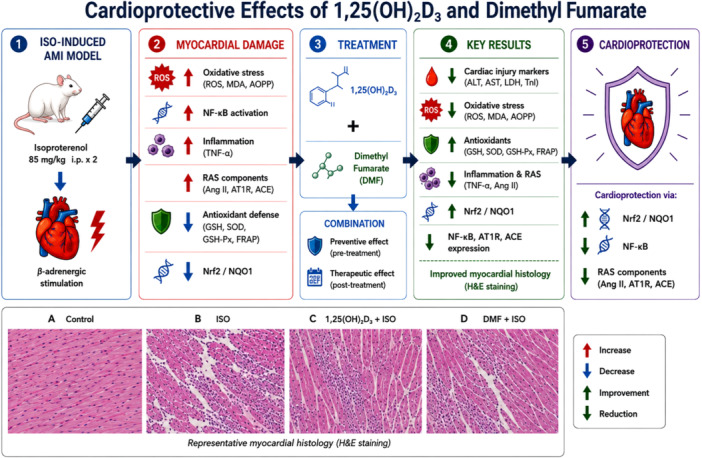

**Abbreviations**
1,25(OH)_2_D_3_
: 1,25‐Dihydroxycholecalciferol (active form of vitamin D)
ACE: Angiotensin‐Converting Enzyme
ALT: Alanine Aminotransferase
AMI: Acute Myocardial Infarction
Ang II: Angiotensin II
AOPP: Advanced Oxidation Protein Products
AST: Aspartate Aminotransferase
AT1R: Angiotensin II Type 1 Receptor
CAT: Catalase
DMF: Dimethyl Fumarate
FRAP: Ferric Reducing Antioxidant Power
GSH: Glutathione
GSH‐Px: Glutathione Peroxidase
ISO: Isoproterenol
LDH: Lactate Dehydrogenase
MDA: Malondialdehyde
NF‐κB: Nuclear Factor kappa B
NQO1: NAD(P)H Quinone Dehydrogenase 1
Nrf2: Nuclear Factor Erythroid 2‐Related Factor 2
RAS: Renin–Angiotensin System
ROS: Reactive Oxygen Species
SOD: Superoxide Dismutase
TGF‐β: Transforming growth factor‐beta
TNF‐α: Tumor Necrosis Factor‐alpha

## 1 Introduction

Isoproterenol (ISO) is a synthetic β‐adrenergic agonist, and it is used frequently to induce experimental acute myocardial injury (AMI). It produces a clinical picture similar to that seen in humans [1, 2, 3]. Exposure to isoproterenol (ISO) in rodent models has been associated with elevated oxidative stress within the cardiac tissue [3, 4, 5, 6, 7, 8, 9, 10]. This effect is characterized by increased reactive oxygen and nitrogen species (ROS/RNS), lipid peroxides, and various protein oxidation products. Furthermore, ISO acts to suppress the gene and protein expression of Nrf2 (nuclear factor erythroid 2‐dependent factor), alongside its downstream antioxidant proteins and enzymes, specifically heme oxygenase 1 (HO‐1), NADPH‐quinone oxidoreductase (NQO1), superoxide dismutase (SOD), and glutathione peroxidase (GSH‐Px) [3, 4, 5, 6, 7, 8, 9, 10]. Concurrently, ISO induces inflammation by elevating NF‐κB (nuclear factor‐kappa B), a central inflammatory transcription factor, and boosting pro‐inflammatory cytokines, notably tumor necrosis factor (TNF‐α) and interleukin 6 (IL‐6) [3, 4, 5, 6, 7, 8, 9, 10]. This cascade of oxidative and inflammatory events culminates in increased cellular apoptosis and necrosis throughout the heart.

The renin‐angiotensin system (RAS), which is crucial for regulating blood pressure and water‐electrolyte balance, is expressed across numerous organs, including the heart [11]. The system comprises two axes‐the classical and the alternative‐that mediate opposing biological effects. The classical axis uses angiotensin‐converting enzyme (ACE) to generate angiotensin II (Ang II), the primary effector peptide in this pathway. Ang II acts by binding to the angiotensin II type 1 receptor (AT1R), resulting in increased vascular contractility, oxidative stress and inflammation, and the promotion of proliferative and fibrotic changes. Conversely, the alternative RAS axis provides a counter‐regulatory balance. This axis is defined by ACE2, which generates angiotensin‐(1–7) [Ang‐(1–7)] from Ang II. Ang‐(1–7) acts via the Mas receptor (MasR) and is known for its vasodilator, anti‐inflammatory, antiproliferative, and antifibrotic properties [11].

Extensive research indicates that RAS activation is a major contributor to myocardial damage [12, 13]. Its pathological influence stems from its ability to stimulate ROS formation and subsequent oxidative stress. This activation also results in elevated concentrations of inflammatory mediators (TNF‐α and IL‐6) and the profibrotic cytokine TGF‐β (transforming growth factor‐beta). Consequently, this heightened RAS activity creates a detrimental cycle that further accelerates oxidative stress and inflammation while initiating fibrotic changes in the tissue [12, 13]. Supporting these findings, studies on coronary artery ligation‐induced AMI have detected increased levels of Ang II and elevated protein expression of AT1R and ACE in serum and heart tissue, but ACE2 expression decreased in the coronary arteries [14]. Furthermore, plasma and heart tissue from rats with ISO‐induced AMI exhibit increased ACE activity [15].

The ISO‐induced myocardial injury model is widely used by researchers to elucidate the mechanisms underlying the pathogenesis of acute myocardial infarction (AMI) [3, 4, 5, 6, 7, 8, 9, 10]. Using this model, researchers have focused on identifying agents capable of preventing myocardial damage (preventive agents) [3, 4, 5, 6, 7, 8, 9, 10] and/or addressing existing damage (therapeutic agents) [16, 17, 18, 19, 20]. This crucial research context involves evaluating the efficacy of diverse compound classes, including antioxidants, anti‐inflammatories, Nrf2 activators, RAS inhibitors, and calcium antagonists [2, 3].

The biologically active form of vitamin D, 1,25‐dihydroxycholecalciferol [1,25(OH)_2_D_3_] (calcitriol), mediates its effects via the VDR (Vitamin D receptor) [21, 22, 23]. This hormone is well known for its multifaceted protective roles, including antioxidant, anti‐inflammatory, and antiapoptotic activities. At the cellular level, calcitriol is reported to exert its benefits by activating the Nrf2 signaling pathway while concurrently inhibiting the RAS [21, 22, 23]. Due to this unique combination of properties, calcitriol provides significant protective effects across numerous tissues and organs, including vital cardiovascular functions [21, 22, 23].

Targeting the Nrf2 system has emerged as a promising preventive and therapeutic strategy for mitigating oxidative stress [24, 25]. Various compounds, including numerous phytochemicals (such as sulforaphane, isoflavones, and polyphenols) and several synthetic agents, have been identified as Nrf2 activators [26]. Among these, dimethyl fumarate (DMF) is a synthetic activator recognized for its antioxidant, anti‐inflammatory, and immunomodulatory properties. DMF is approved for clinical use in the treatment of conditions such as psoriasis and multiple sclerosis. Its mechanism involves activating the Nrf2‐ARE (antioxidant‐responsive element) system, which subsequently drives the expression of protective antioxidant enzymes and proteins. Moreover, DMF exerts anti‐inflammatory effects by inhibiting NF‐κB nuclear translocation, thereby suppressing NF‐κB‐induced transcription of proinflammatory cytokines. Due to its combined antioxidant and anti‐inflammatory properties, DMF has been reported to be highly effective in protecting various tissues, including the cardiovascular system [27, 28].

Research gaps persist regarding the combined and individual effects of vitamin D specifically 1,25(OH)_2_D_3_) and the Nrf2 activator DMF in the ISO‐induced AMI model. While some reports 1,25(OH)_2_D_3_ [29] and the vitamin D receptor agonist paricalcitol [30] as pretreatments in this model, no published work has investigated the efficacy of DMF in ISO‐induced AMI. However, some reports indicate that DMF is effective in ISO‐induced cardiac hypertrophy [31] and in coronary artery occlusion‐induced AMI [32, 33]. On the other hand, 1,25(OH)_2_D_3_ has been reported to be more effective with a synergistic effect when used in combination with other Nrf2 activators such as sulforaphane [34] or resveratrol [35]. Therefore, in the current study, it might be interesting to demonstrate the existence of a synergistic interaction between 1,25(OH)_2_D_3_ and DMF. For this reason, we aimed to investigate the preventive potential of 1,25(OH)_2_D_3_ and DMF, administered individually or in combination against ISO‐induced AMI in rats. Moreover, we assessed the therapeutic efficacy of 1,25(OH)_2_D_3_ plus DMF when administered as a post‐treatment regimen. To fully characterize these effects, we quantified cardiac damage markers in serum, assessed cardiac histopathology, and measured markers of ROS formation, the prooxidant/antioxidant balance, Nrf2/NQO1 and NF‐κB mRNA expression, AT1R and ACE mRNA expressions, and serum Ang II levels.

## 2 Materials and Methods

### 2.1 Animals

Male Sprague–Dawley rats weighing 180–200 g were acquired from the Istanbul University, Aziz Sancar Institute of Experimental Medicine (DETAE). The animals were maintained under controlled environmental conditions (24°C–26°C, 12‐h light/dark cycle) in stainless‐steel cages, housing three to four rats per cage. Standard laboratory chow and water were provided ad libitum. All experimental protocols adhered to institutional ethical guidelines and received approval from the Istanbul University Animal Experiments Local Ethics Committee (Approval Date: 25/05/2023; Approval Number: 1778536). The rats were subsequently randomly assigned into nine experimental groups.

a. Control group (*n* = 6): Rats received standard rat chow and drinking water.
b. 1,25(OH)_2_D_3_ group (*n* = 6): A total of 4 doses of 1,25(OH)_2_D_3_ (1 µg/kg, by i.p. injection) on Days 1, 3, 5, 7 were administered to rats during the experiment.
c. DMF group (*n* = 6): DMF (50 mg/kg/day, by gavage) was administered for 10 days.
d. 1,25(OH)_2_D_3_ + DMF group (*n *= 6): A total of 4 doses of 1,25(OH)_2_D_3_ (1 µg/kg, i.p.), and DMF (50 mg/kg/day, gavage) were administered for 10 days.
e. ISO group (*n* = 8): Rats received DMF solvent (1% dimethyl sulfoxide, gavage) and 1,25(OH)_2_D_3_ solvent (% 0.9% NaCl, i.p.) for 10 days, followed by ISO (85 mg/kg, by i.p. injection) on the 9th and 10th days.
  (f–h) As in the relevant pretreatment groups (1,25(OH)_2_D_3_, DMF, and 1,25(OH)_2_D_3_ + DMF), rats received the same 10‐day pretreatment protocols before ISO. ISO (85 mg/kg, i.p.) was administered on Days 9 and 10 in the following groups (*n* = 7, each):
f. 1,25(OH)_2_D_3_ + ISO group (*n* = 7).
g. DMF + ISO group (*n* = 7).
h. 1,25(OH)_2_D_3_ + DMF + ISO group (*n* = 7).
i. ISO + 1,25(OH)_2_D_3_ + DMF group (*n* = 7): Rats were administered ISO (85 mg/kg; i.p. injection) on Days 1 and 2. Starting on Day 3, they were administered DMF (50 mg/kg; gavage) for 10 days and four doses of 1,25(OH)_2_D_3_ (1 µg/kg; i.p. injection) every other day.

ISO was dissolved in physiological saline, and DMF was dissolved in 1% dimethyl sulfoxide (DMSO); these vehicles were also applied to the control and ISO groups, as described in the experimental procedure. 1,25(OH)_2_D_3_ and DMF were administered 1 h before ISO and at ½‐hour intervals.

The doses and routes of administration of ISO [6, 7, 8, 9, 10], 1,25(OH)_2_D_3_ [29, 36], and DMF [37] used in our study were based on previous studies.

### 2.2 Blood and Tissue Samples

At the conclusion of the treatment periods, all rats were subjected to overnight fasting and subsequently euthanized on Day 11 (Day 13 for Group 9) under sevoflurane anesthesia.

Blood samples were collected from the retro‐orbital venous plexus using sterile capillary tubes. Blood samples were centrifuged at 1500 × *g* for 10 min to separate the serum. Heart tissues were quickly removed and washed with 0.9% NaCl. The heart tissue was homogenized in 0.15 M KCl (10%; w/v) and centrifuged at 600 × *g* for 10 min, and the supernatant was used for biochemical analyses. Tissues and samples were stored at −80°C until experiments were performed. Some tissues were placed in formalin for histopathological examination.

### 2.3 Serum Analyses

Serum activities of Alanine Aminotransferase (ALT), Aspartate Aminotransferase (AST), and Lactate Dehydrogenase (LDH) were quantified using a Cobas Integra autoanalyzer (Roche Diagnostics, Mannheim, Germany). These measurements were performed at the laboratory of the Istanbul Medical Faculty Clinical Biochemistry Center. High‐sensitivity Troponin I (hs Troponin I) levels were measured separately using a commercially available ELISA kit.

### 2.4 Heart Tissue Analyses

1. Ang II levels were measured using an Angiotensin II ELISA kit (CEA005Ra, Cloud‐Clone Corp., USA) according to the manufacturer's instructions.
2. Measurement of ROS and MDA levels
  ROS levels were measured fluorometrically using a previously described method. Tissue homogenates were incubated with one mM 2,7‐dichlorodihydrofluorescein diacetate (DCFH‐DA) at 37°C for 30 min. The resulting 2,7‐dichlorodihydrofluorescein was read on a fluorometric microplate reader (*λ*_excitation_: 485 nm, *λ*_emission_: 538 nm). Results were given as relative fluorescence units (RFU) [38].
  The complex formed by MDA, one of the end products of lipid peroxidation, with thiobarbituric acid (TBA) was measured spectrophotometrically. Results were calculated using the extinction coefficient (*ε* = 1.56 × 10^−5^ M^−1^ cm^−1^) and reported as nmol/mg protein [39].
3. Measurement of advanced oxidation products (AOPP) of proteins
  The absorbance of heart homogenates in an acidic citric acid medium was measured at 340 nm. The results were compared by standardizing with triiodide, prepared by oxidizing potassium iodide with chloramine‐T [40].
4. Measurement of GSH and FRAP levels
  Ferric Reducing Antioxidant Power (FRAP) and Glutathione (GSH) levels were both measured in the heart homogenates. The FRAP levels, which reflect the overall antioxidant potential, were determined using the standard methodology initially described by Benzie and Strain [41]. GSH levels were determined spectrophotometrically using 5,5′‐dithiobis‐2‐nitrobenzoic acid (DTNB) as the substrate, following the method described by Beutler et al. [42].
5. SOD and GSH‐Px activities
  SOD and GSH‐Px enzyme activities were quantified in the post‐mitochondrial fractions. These fractions were obtained after centrifuging the heart homogenate at 10,000 × *g* for 20 min at 4°C. SOD activity was measured by the ability of riboflavin‐sensitized ο‐dianisidine to increase the photooxidation rate [43]. GSH‐Px activity was measured using cumene hydroperoxide as the substrate, according to the procedure detailed by Lawrence and Burk [44].
6. Protein determination
  The protein content of post‐mitochondrial fractions and tissue homogenates was determined using the bicinchoninic acid method [45].
7. Nrf2, NQO1, NF‐κB, AT1R, and ACE Gene Expression Analyses in Heart Tissue

### 2.5 RNA Isolation and Quality Control

Total RNA was isolated from the collected heart tissues using a commercial kit (Qiagen RNeasy, Germany). RNA purity and quantity were subsequently assessed spectrophotometrically utilizing a NanoDrop system (Thermo Scientific, USA).

### 2.6 cDNA Synthesis and PCR Setup

cDNA was synthesized from the isolated RNA samples using a reverse transcriptase enzyme kit (High‐Capacity cDNA Reverse Transcription Kit, Applied Biosystems, USA). qRT‐PCR analyses were then conducted on the 7500 Fast Real‐Time PCR System (Applied Biosystems, USA) with SYBR Green Master Mix (Applied Biosystems, USA). Gene‐specific QuantiTect Primer Assays (Qiagen, Germany) were employed for each target gene as follows: Nrf2 (GeneGlobe ID: PPR45094A), NQO1 (GeneGlobe ID: PPR45314A), NF‐κB (GeneGlobe ID: PPR42746A), AT1R (GeneGlobe ID: PPR44498A), and ACE (GeneGlobe ID: PPR44697A). All qRT‐PCR reactions were performed using the QuantiTect SYBR Green PCR Kit (Cat. No. 330001, Qiagen, Germany).

### 2.7 Thermal Cycling and Data Analysis

The PCR thermal cycling conditions included an initial denaturation step at 95°C for 10 min, followed by 40 cycles of denaturation at 95°C for 15 s and a combined annealing/extension phase at 60°C for 1 min. Gene expression levels were calculated using the 2^−^^ΔΔCt^ method, utilizing beta‐actin as the internal reference gene.

8. Histopathological Examinations:

For histopathological examination, tissues were fixed in 10% formaldehyde. After routine automated tissue tracking, the tissues were embedded in paraffin. 3 μm‐thick sections obtained from each paraffin block were stained with hematoxylin and eosin (H&E). Myocardial injury was assessed based on characteristic features of early myocyte damage, including hypereosinophilic fibers indicative of necrosis, interstitial hemorrhage, edema, disruption of myofiber integrity, and inflammatory cell infiltration. The proportion of each parameter relative to the normal myocardial tissue was determined as a percentage. Based on the extent of these findings, involvement was classified as mild (0%–20%), moderate (21%–50%), or severe (51%–100%)

For immunohistochemical evaluation, 3‐μm‐thick paraffin sections were deparaffinized, rehydrated, and subjected to heat‐induced antigen retrieval. After blocking endogenous peroxidase activity, sections were incubated overnight at 4°C with primary antibodies against NQO1 (1:100, Cat# DF6437, Affinity Biosciences), Nrf2 (1:100, Cat# AF0639, Affinity Biosciences), and NF‐κB (1:100, Cat# AF5006, Affinity Biosciences). Following incubation with an appropriate secondary antibody, immunoreactivity was visualized using a DAB‐based chromogenic detection system and sections were counterstained with hematoxylin. Immunostaining was evaluated by a pathologist blinded to the experimental groups. The percentage of positively stained cells was assessed semiquantitatively and classified as mild (0%–20%), moderate (21%–50%), or marked (51%–100%).

### 2.8 Statistical Analysis

Statistical analyses were conducted using SPSS software (version 21.0; SPSS Inc., Chicago, IL, USA). All quantitative data are presented as mean ± standard deviation (SD). The normality of data distribution was assessed using the Kolmogorov–Smirnov test, and homogeneity of variances was evaluated using Levene's test. For normally distributed data, one‐way ANOVA followed by Tukey's HSD post hoc test was used for multiple comparisons. For non‐normally distributed data, the Kruskal–Wallis test followed by the Mann–Whitney U post hoc test was applied. A *p*‐value < 0.05 was considered statistically significant. Additional significance levels were indicated as *p* < 0.01 and *p* < 0.001. Outlier analysis was performed using the Grubbs' test; no statistically significant outliers were identified. Data distribution was visually assessed using scatter plots of individual data points.

## 3 Results

### 3.1 Body Weight, Heart Weight, and Cardiac Index

Body weight, heart weight, and cardiac index (heart weight × 100/body weight) values did not change when 1,25(OH)_2_D_3_, DMF, and 1,25(OH)_2_D_3_ + DMF were administered to rats in the control groups.

Body weight decreased, heart weight remained unchanged, but cardiac index increased significantly in ISO‐treated rats compared with the control group. Significant decreases in cardiac index were observed in ISO‐applied rats following pre‐ and post‐treatment with 1,25(OH)_2_D_3_ + DMF compared with the ISO group (Table 1).

**Table 1:** Body weight, heart weight, and heart index in the control and experimental groups.

Groups (*n*)	Body weight (g)	Heart weight (g)	Heart index (%)
Control (*n* = 6)	311.6 ± 30.1	1.13 ± 0.11	0.36 ± 0.03
1,25(OH)_2_D_3_ (1 μg/kg) (*n* = 6)	318.8 ± 22.8	1.02 ± 0.15	0.33 ± 0.03
DMF (*n* = 6)	280.8 ± 0.18	0.89 ± 0.07	0.31 ± 0.01
1,25(OH)_2_D_3_ + DMF (*n* = 6)	324.5 ± 19.6	1.21 ± 0.19	0.37 ± 0.04
ISO (*n* = 8)	251.5 ± 13.2	1.20 ± 0.21	0.48 ± 0.10^a^
1,25(OH)_2_D_3_ + ISO (*n* = 7)	289.0 ± 22.1	1.22 ± 0.35	0.41 ± 0.10
DMF + ISO (*n* = 7)	268.4 ± 13.6	1.21 ± 0.14	0.45 ± 0.05^a^
1,25(OH)_2_D_3_ + DMF + ISO (*n* = 7)	279.7 ± 39.8	1.09 ± 0.19	0.39 ± 0.04^b^
ISO + 1,25(OH)_2_D_3_ + DMF (*n* = 7)	298.0 ± 9.03^b^	1.03 ± 0.08	0.34 ± 0.02^b^

*Note:* Control and ISO‐Induced rats treated with 1,25(OH)_2_D_3_, DMF, and their combination.

Data are expressed as mean ± SD.

^a^ *p* < 0.05 versus control group.

^b^ *p* < 0.05 versus ISO group.

### 3.2 Biochemical Findings

**Figure 1:**
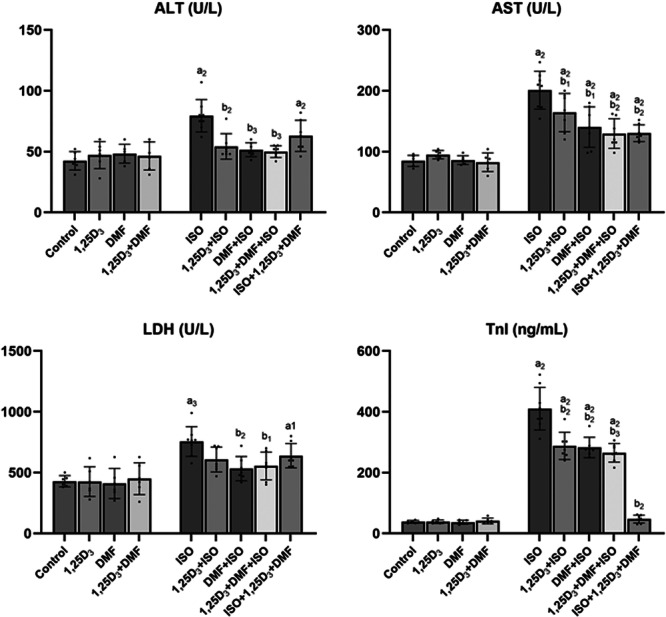
Effects of 1,25(OH)_2_D_3_, DMF, and their combination on serum cardiac injury markers in rats with ISO‐induced AMI. Serum levels of alanine aminotransferase (ALT, U/L), aspartate aminotransferase (AST, U/L), lactate dehydrogenase (LDH, U/L), and troponin I (TnI, ng/mL) are presented for Control, 1,25(OH)_2_D_3_, DMF, 1,25(OH)_2_D_3_ + DMF, ISO, ISO + 1,25(OH)_2_D_3_, ISO + DMF, and ISO + 1,25(OH)_2_D_3_ + DMF groups. Data are expressed as mean ± SD (*n* = 7 per group). ^a^ *p* < 0.05 versus Control group; ^b^ *p* < 0.05 versus ISO group. Subscripts indicate levels of significance: _1_ *p* < 0.05, _2_ *p* < 0.01, and _3_ *p* < 0.001. Statistical analyses were performed as described in the Statistical Analysis section.

Serum ALT, AST, LDH activities, and TnI I levels were significantly increased in the ISO group compared to the control group. In AMI rats, 1,25(OH)_2_D_3_ and DMF pre‐treatments, alone and in combination, significantly reduced serum cardiac damage markers compared with the ISO group. However, no significant decrease was observed in serum LDH activity in the 1,25(OH)_2_D_3_ + ISO group. A significant reduction in AST activity and TnI levels was observed after post‐treatment with 1,25(OH)_2_D_3_ + DMF in rats with ISO‐induced AMI. Moreover, TnI levels in this group returned to normal levels (Figure 1).

### 3.3 Cardiac Oxidative Stress Parameters

**Figure 2:**
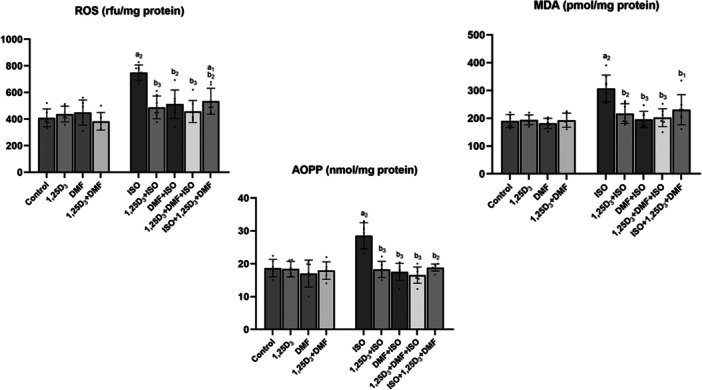
Effects of 1,25(OH)_2_D_3_, DMF, and their combination on cardiac oxidative stress markers in rats with ISO‐induced AMI. Cardiac levels of reactive oxygen species (ROS, RFU/mg protein), malondialdehyde (MDA, pmol/mg protein), and advanced oxidation protein products (AOPP, nmol/mg protein) are presented for Control, 1,25(OH)_2_D_3_, DMF, 1,25(OH)_2_D_3_ + DMF, ISO, ISO + 1,25(OH)_2_D_3_, ISO + DMF, and ISO + 1,25(OH)_2_D_3_ + DMF groups. Data are expressed as mean ± SD. ^a^ *p* < 0.05 versus Control group; ^b^ *p* < 0.05 versus ISO group. Subscripts indicate levels of significance: _1_ *p* < 0.05, _2_ *p* < 0.01, and _3_ *p* < 0.001. Statistical analyses were performed as described in the Statistical Analysis section.

In normal rats, administration of 1,25(OH)_2_D_3_, DMF, or 1,25(OH)_2_D_3_ + DMF did not cause significant changes in ROS, MDA, or AOPP levels in heart tissue. However, cardiac ROS, MDA, and AOPP levels were significantly higher in rats with ISO‐induced AMI than in controls. In the 1,25(OH)_2_D_3_ + ISO, DMF + ISO, and 1,25(OH)_2_D_3_ + DMF + ISO groups, these prooxidant parameters decreased significantly compared to the ISO group (*p* < 0.05). Statistically significant decreases were also observed in cardiac ROS, MDA, and AOPP levels in rats with AMI following post‐treatment with 1,25(OH)_2_D_3_ plus DMF (Figure 2).

### 3.4 Cardiac Antioxidant Parameters

**Figure 3:**
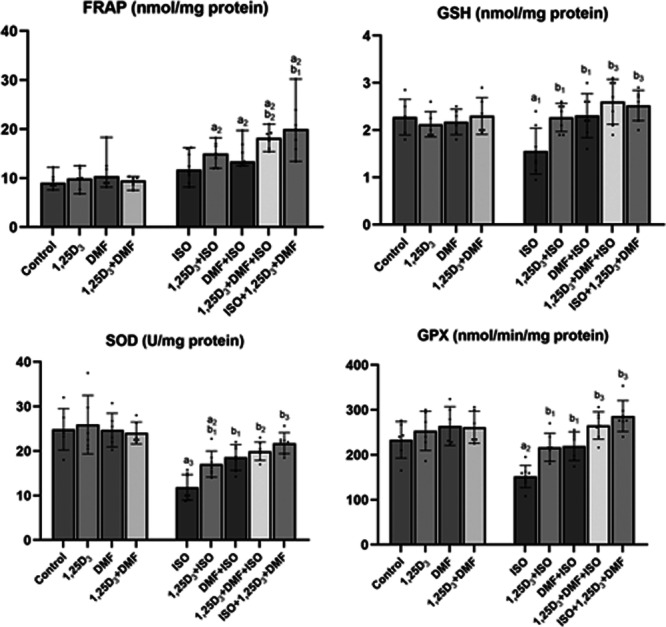
Effects of 1,25(OH)_2_D_3_, DMF, and their combination on cardiac oxidative stress parameters in rats with ISO‐induced AMI. Cardiac levels of ferric reducing antioxidant power (FRAP, nmol/mg protein), reduced glutathione (GSH, nmol/mg protein), superoxide dismutase (SOD, U/mg protein), and glutathione peroxidase (GPX, nmol/min/mg protein) are presented for Control, 1,25(OH)_2_D_3_, DMF, 1,25(OH)_2_D_3_ + DMF, ISO, ISO + 1,25(OH)_2_D_3_, ISO + DMF, and ISO + 1,25(OH)_2_D_3_ + DMF groups. Data are expressed as mean ± SD (*n* = 7 per group). ^a^ *p* < 0.05 versus Control group; ^b^ *p* < 0.05 versus ISO group. Subscripts indicate levels of significance: _1_ *p* < 0.05, _2_ *p* < 0.01, and _3_ *p* < 0.001. Statistical analyses were performed as described in the Statistical Analysis section.

In control groups, pretreatments of 1,25(OH)_2_D_3_, DMF, and 1,25(OH)_2_D_3_ + DMF alone did not cause significant changes in antioxidant parameters. ISO administration caused a significant decrease in GSH levels and SOD and GPX activities and an increase in FRAP values compared to the control group. Contrarily, 1,25(OH)_2_D_3_, DMF, and 1,25(OH)_2_D_3_ + DMF treatments caused significant increases in GSH levels and SOD and GPx activities, which were already decreased in rats with myocardial infarction. These treatments also significantly increased FRAP values in ISO‐applied rats as compared to controls. Significant increases in GSH and FRAP levels and SOD and GPX activities were also found in rats with AMI due to 1,25(OH)_2_D_3_ + DMF posttreatment (Figure 3).

### 3.5 Cardiac Ang II and TNF‐α Levels

**Figure 4:**
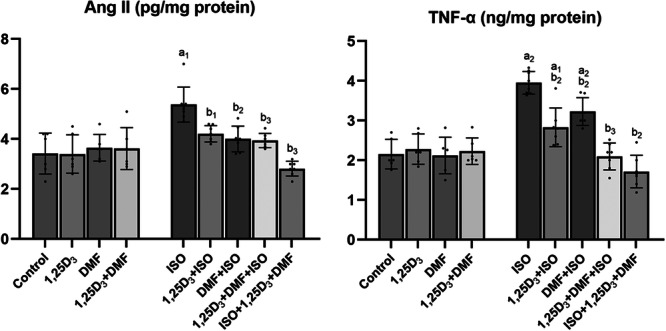
Effects of 1,25(OH)_2_D_3_, DMF, and their combination on cardiac angiotensin II (Ang II) and TNF‐α levels in rats with ISO‐induced AMI. Cardiac Ang II (pg/mg protein) and TNF‐α (ng/mg protein) levels are presented for Control, 1,25(OH)_2_D_3_, DMF, 1,25(OH)_2_D_3_ + DMF, ISO, ISO + 1,25(OH)_2_D_3_, ISO + DMF, and ISO + 1,25(OH)_2_D_3_ + DMF groups. Data are expressed as mean ± SD. ^a^ *p* < 0.05 versus Control group; ^b^ *p* < 0.05 versus ISO group. Subscripts indicate levels of significance: _1_ *p* < 0.05,_2_ *p* < 0.01, and _3_ *p* < 0.001. DMF, dimethyl fumarate; ISO, isoproterenol; 1,25(OH)_2_D_3_, 1,25‐dihydroxyvitamin D_3_; Ang II, angiotensin II; TNF‐α, tumor necrosis factor‐α. Statistical analyses were performed as described in the Statistical Analysis section.

Ang II and TNF‐α levels were significantly increased in heart tissue from rats with AMI compared with the control group. Significant decreases in cardiac Ang II and TNF‐α levels were observed in AMI rats following 1,25(OH)_2_D_3_ and DMF pretreatment. Moreover, pre‐ and post‐treatment with 1,25(OH)_2_D_3_ plus DMF in rats with AMI resulted in significant decreases in Ang II and TNF‐α levels compared with the ISO group (Figure 4).

### 3.6 Nrf2, NQO1, NF‐κB, AT1R, and ACE mRNA Expressions

**Figure 5:**
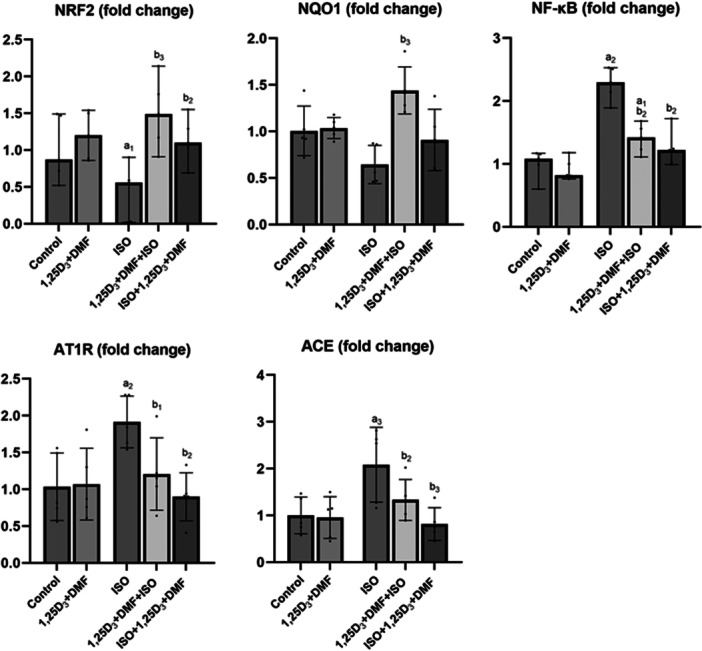
Gene expression levels of Nrf2, NQO1, NF‐κB, AT1R, and ACE in experimental groups. Gene expression levels are presented as fold change relative to the Control group. Data are expressed as mean ± SD. Groups: Control, 1,25D_3_ + DMF, ISO, 1,25D_3_ + DMF + ISO, and ISO + 1,25D_3_ + DMF. ^a^ Statistically significant difference compared with the Control group; ^b^statistically significant difference compared with the ISO group. Subscripts indicate significance levels: _1_ *p* < 0.05, _2_ *p* < 0.01, and _3_ *p* < 0.001. ISO, isoproterenol; DMF, dimethyl fumarate; 1,25D_3_, 1,25‐dihydroxyvitamin D_3_; Nrf2, nuclear factor erythroid 2‐related factor 2; NQO1, NAD(P)H quinone oxidoreductase 1; NF‐κB, nuclear factor kappa B; AT1R, angiotensin II type 1 receptor; ACE, angiotensin‐converting enzyme. Statistical analyses were performed using one‐way ANOVA followed by Tukey's post hoc test for normally distributed data and the Kruskal–Wallis test followed by Mann–Whitney *U* post hoc comparisons for non‐normally distributed data. Statistical analyses were performed as described in the Statistical Analysis section.

In this study, the effects of 1,25(OH)_2_D_3_ + DMF pretreatment and posttreatment on Nrf2, NQO1, NF‐κB, AT1R, and ACE mRNA expression in rats with AMI were also determined. According to the results, cardiac mRNA expression of Nrf2, NQO1, NF‐κB, AT1R, and ACE did not change in normal rats following pretreatment with 1,25(OH)_2_D_3_ + DMF. However, ISO administration decreased Nrf2 and NQO1 mRNA expression in rat cardiac tissue, but decreases in NQO1 mRNA were not significant. Cardiac NF‐κB, AT1R, and ACE mRNA expression was significantly increased by ISO application, in the preventive and therapeutic groups with myocardial injury treated with 1,25(OH)_2_D_3_ plus DMF. Nrf2 and NQO1 mRNA expression increased. In contrast, NF‐κB, AT1R, and ACE mRNA expression decreased significantly compared with the ISO group. However, the increase in NQO1 mRNA expression in the therapeutic group was not statistically significant (Figure 5).

### 3.7 Histopathological Assessment of Myocardial Damage

Histopathological evaluation revealed normal myocardial architecture in the control group and in the groups treated with 1,25(OH)_2_D_3_ or dimethyl fumarate (DMF) alone. The ISO‐administered group demonstrated marked myocardial injury, characterized by hypereosinophilic and degenerating myocytes, disruption of fiber continuity, interstitial edema, hemorrhage, and prominent inflammatory cell infiltration. Myocardial injury was reduced in both the 1,25(OH)_2_D_3_ + ISO and DMF + ISO groups when compared with the ISO group. These groups exhibited less pronounced myocyte degeneration and reduced inflammatory cell infiltration. Notably, injury was less pronounced in the DMF‐treated ISO group, in which ISO‐related alterations persisted, yet overall myocardial structure was better preserved.

**Figure 6:**
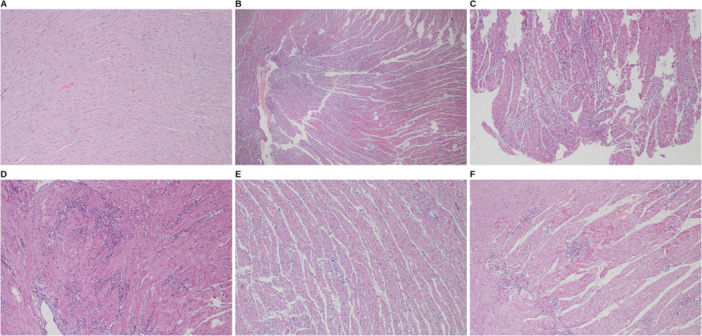
Histopathological evaluation of myocardial tissue in treated and control groups. (A) Control, DMF, and 1,25(OH)_2_D_3_ groups showed normal myocardial architecture with regularly arranged cardiac muscle fibers and intact cardiomyocytes, without evidence of necrosis or inflammatory cell infiltration (H&E, ×100). (B) The ISO group exhibited extensive myocardial necrosis, loss of cardiomyocyte integrity, and dense inflammatory cell infiltration (H&E, ×100). (C) The 1,25(OH)_2_D_3_ + ISO group showed focal myocardial necrosis accompanied by marked inflammatory cell infiltration (H&E, ×100). (D) The DMF + ISO group demonstrated largely preserved myocardial architecture with mild‐to‐moderate interstitial inflammatory cell infiltration (H&E, ×100). (E) The DMF + 1,25(OH)_2_D_3_ + ISO group revealed interstitial edema with mild inflammatory cell infiltration and predominantly preserved myocardial architecture (H&E, ×100). (F) The ISO + 1,25(OH)_2_D_3_ + DMF group showed focal myocardial necrosis associated with moderate inflammatory cell infiltration (H&E, ×100).

In the pretreated ISO group, myocardial alterations were milder than in the ISO group, showing reduced inflammatory infiltration and partially preserved myocardial architecture. However, posttreatment with 1,25(OH)_2_D_3_ + DMF in ISO‐applied rats, myocardial structure was better preserved than in the ISO group, inflammatory findings were milder, and myofiber organization appeared more regular, indicating a supportive therapeutic effect (Figure 6).

### 3.8 Immunohistochemical Expression of NQO1, NF‐κB, and Nrf2

**Figure 7:**
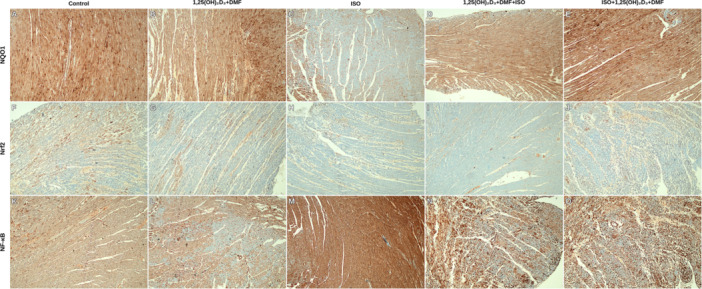
Immunohistochemical staining for NQO1 (A–E), Nrf2 (F–J), and NF‐κB (K–O) in myocardial tissue. Representative photomicrographs (×100 magnification) of the Control, 1,25(OH)_2_D_3_ + DMF, ISO, 1,25(OH)_2_D_3_ + DMF + ISO (pretreatment), and ISO + 1,25(OH)_2_D_3_ + DMF (post‐treatment) groups. NQO1 and Nrf2 immunostaining were both reduced in the ISO group compared with Control and increased following combination pretreatment and post‐treatment, whereas NF‐κB immunostaining showed the opposite pattern, with marked upregulation in the ISO group and reduced expression in the combination‐treated groups.

Immunohistochemical findings were consistent with the histopathological and mRNA expression results. NQO1 immunoreactivity was strong in the Control and 1,25(OH)_2_D_3_ + DMF groups, whereas it was reduced in the ISO group; both 1,25(OH)_2_D_3_ + DMF pretreatment and post‐treatment increased NQO1 immunostaining compared with the ISO group. NF‐κB immunoreactivity was minimal in the Control and 1,25(OH)_2_D_3_ + DMF groups, markedly increased in the ISO group—consistent with the interstitial edema and fibrotic changes observed on histopathological examination—and was reduced in both combination‐treated groups relative to the ISO group. Similarly, Nrf2 immunoreactivity was strong in the Control and 1,25(OH)_2_D_3_ + DMF groups, markedly reduced in the ISO group, and increased in both the pretreatment and post‐treatment combination groups relative to the ISO group (Figure 7).

## 4 Discussion

ISO, a synthetic catecholamine and beta‐adrenergic agonist, is widely utilized to establish AMI models. Consistent with previous literature, ISO was administered in our study at a dose of 85 mg/kg in two doses, separated by 24 h [4, 5, 6, 7, 8, 9, 10]. Confirmation of myocardial damage in ISO‐administered rats was based on multiple indicators, thereby demonstrating successful AMI induction. Specifically, we observed significant increases in serum enzyme activities (ALT, AST, and LDH) and troponin I levels, all established indicators of cardiac injury, which were further substantiated by histopathological findings [4, 5, 6, 7, 8, 9, 10].

Studies have consistently demonstrated that ISO application increases oxidative damage in cardiac tissue, as evidenced by elevated ROS formation, lipid and protein oxidation products, and subsequent DNA damage [3, 4, 5, 6, 7, 8, 9, 10]. Concurrently, suppression of the Nrf2 system is observed, indicated by decreased gene expression [4, 5, 9] and protein levels [6, 7, 8, 9, 10] in the hearts of ISO‐treated rodents. Nrf2 is recognized as the master regulatory protein of the antioxidant system. Under basal conditions, its activity is tightly controlled: Nrf2 is sequestered in the cytosol by binding to its inhibitor, Keap1 (Kelch‐like ECH‐associated protein 1), and its levels are maintained at low levels via ubiquitination and subsequent proteasomal degradation. However, elevated ROS levels trigger a conformational change in the Nrf2‐Keap1 complex, driven by oxidation of Keap1's sulfhydryl groups. This molecular alteration allows Nrf2 to dissociate from Keap1, translocate to the nucleus, and bind to Antioxidant Response Elements (AREs), thereby initiating the synthesis of various protective antioxidant molecules and proteins [24, 25, 26].

Our investigation confirms that ISO‐induced AMI resulted in a pronounced shift toward an oxidative state in the cardiac tissue. We detected significant increases in several pro‐oxidative markers, including cardiac ROS formation, MDA levels (reflecting lipid peroxidation), and AOPP levels (indicating protein oxidation). This pro‐oxidant surge was accompanied by a failure of the endogenous defense system: specifically, we observed reduced GSH levels and reduced SOD and GSH‐Px activities. Furthermore, the core defense mechanism was suppressed, evidenced by decreased mRNA expressions of Nrf2 and its essential target, NQO1. Overall, the data clearly demonstrate a severe disruption of the prooxidant‐antioxidant equilibrium in the hearts of ISO‐treated rats, leading to significant oxidative stress, consistent with findings reported in the existing literature [3, 4, 5, 6, 7, 8, 9, 10]. The variability observed in certain parameters likely reflects the biological heterogeneity inherent in the ISO‐induced AMI model.

NF‐κB is a key transcription factor for cardiovascular health and disease. It is involved in the release of proinflammatory cytokines and the apoptosis of cardiomyocytes. Inhibition of NF‐κB has been suggested to be effective in acute hypoxia and reperfusion injury [46]. ISO administration has been reported to increase NF‐κB and proinflammatory cytokine gene and protein expression in rodent hearts [4, 6, 7, 9, 10]. In our study, increases in NF‐κB mRNA expression and TNF‐α levels in the heart tissue of the ISO‐treated group indicate the development of heart tissue inflammation.

Increased serum Ang II levels and cardiac AT1R and ACE mRNA expressions were detected in ISO‐administered rats [14]. In addition, pre‐treatment of the AT1R antagonist losartan [46], the renin inhibitor aliskiren [47], the ACE inhibitor enalapril [48], and the ACE2 activator diminazene aceturate (DIZE) [14] were reported to have ameliorative effects on ISO‐induced cardiac damage, demonstrating the importance of the RAS system in the pathogenesis of myocardial infarction. In our study, increased Ang II levels and mRNA expression of AT1R and ACE were observed in the hearts of ISO‐treated rats. These results clearly demonstrate that the classical axis of the RAS system is activated in the infarcted heart.

Our primary investigation centered on determining the protective effects of 1,25(OH)_2_D_3_ and the Nrf2 activator DMF as pretreatments against ISO‐induced myocardial injury. 1,25(OH)_2_D_3_ treatment was reported to reduce cardiac hypertrophy induced by repeated low‐dose ISO [49]. Pretreatment with vitamin D [29] and paricalcitol [30], a VDR activator, has been suggested to be effective in ISO‐induced MI by suppressing cardiac oxidative stress and inflammation. Similar findings were also observed in diabetic rats with myocardial infarction treated with ISO [50]. On the other hand, to our knowledge, no study has directly investigated the effect of the Nrf2 activator DMF on ISO‐induced myocardial injury. However, pre‐administration of DMF has been reported to ameliorate ISO‐induced cardiac hypertrophy at low doses by suppressing oxidative stress and inflammation [31]. Moreover, post‐treatment of DMF in rats undergoing coronary artery ligation has been found to reduce cardiac damage by activating the Nrf2/HO‐1 signaling pathway and suppressing oxidative stress and inflammation [32, 33].

In our study, 1,25(OH)_2_D_3_ pretreatment was found to be beneficial for ISO‐induced cardiac injury, as evidenced by improvements in serum cardiac injury markers (ALT, AST, LDH, and troponin I) and histopathological findings. This treatment was effective in reducing oxidative stress (decreased MDA and AOPP levels, increased GSH levels, and SOD and GSH‐Px activities) and inflammation (decreased TNF‐α levels) in ISO‐induced AMI. Similarly, the current study showed that pre‐administration of DMF, such as 1,25(OH)_2_D_3_, improved these parameters in rats with AMI.

Consistent with the mRNA expression and histopathological findings, immunohistochemical analysis confirmed reduced Nrf2 and NQO1 immunoreactivity and increased NF‐κB immunoreactivity in the myocardium of ISO‐treated rats. The restoration of Nrf2 and NQO1 expression together with attenuated NF‐κB staining following 1,25(OH)_2_D_3_ + DMF treatment provides protein‐level evidence supporting the antioxidant and anti‐inflammatory effects of the combination therapy.

In our study, the preventive and therapeutic potential of the 1,25(OH)_2_D_3_ plus DMF combination was assessed in ISO‐induced AMI. Combination pretreatment was found to increase GSH levels and SOD and GSH‐Px activities, and to decrease oxidative stress parameters and TNF‐α levels in the heart tissue of infarcted rats. Similarly, post‐treatment with the 1,25(OH)_2_D_3_ + DMF combination reduced ISO‐induced myocardial damage, improved the prooxidant–antioxidant balance, and attenuated inflammation. However, when the combination groups were compared with the individual treatment groups (1,25(OH)_2_D_3_ + ISO and DMF + ISO), no statistically significant additional benefit was observed for most parameters examined, except for FRAP and TNF‐α levels. These findings indicate that no significant synergistic interaction exists between these two agents under the present experimental conditions.

On the other hand, in these experimental groups, some mRNA expressions (Nrf2, NF‐κB, ACE, AT1R) were additionally examined. Vitamin D [12, 51, 52] and DMF [33, 53] have been reported to increase Nrf2 expression while suppressing NF‐κB expression in various pathological conditions. On the other hand, vitamin D has been reported to be a negative modulator of the RAS system by activating the ACE2/Ang‐(1–7)/MasR axis and by suppressing the ACE/Ang II/AT1R axis [54].

It is unknown whether DMF has a direct effect on the RAS system. Nevertheless, Nrf2 activation has been shown to prevent Ang II‐induced cardiomyopathy and aortic damage [55, 56], while Nrf2 deficiency exacerbates Ang II‐induced cardiac injury by promoting hypertrophy [57], demonstrating a relationship between the Nrf2 pathway and RAS modulation. Although the precise mechanism underlying this relationship remains unclear, RAS inhibition observed in our study may be related to the suppression of oxidative stress and inflammation mediated by Nrf2 activation.

In our study, pretreatment with a combination of 1,25(OH)_2_D_3_ and DMF elevated Nrf2 and NQO1 mRNA expression, and supressed NF‐κB, AT1R, and ACE mRNA expression and serum Ang II levels. Similarly, post‐treatment with the 1,25(OH)_2_D_3_ + DMF combination increased Nrf2 mRNA expression and decreased NF‐κB, AT1R, and ACE mRNA expression compared with the ISO group.

As a conclusion, our results indicate that (a) pre‐treatments of 1,25(OH)_2_D_3_ and DMF, alone and together, produced a positive ameliorative effect on ISO‐induced myocardial damage, but no synergistic effect was observed, (b) 1,25(OH)_2_D_3_ + DMF combination has a preventive and therapeutic potential for ISO‐induced myocardial damage, (c) this potential of 1,25(OH)_2_D_3_ and DMF in the myocardial infarction may be related to their regulatory activity on oxidative stress, inflammation, Nrf2 and NF‐κB signaling pathways, and RAS.

## Author Contributions

All authors contributed to the conception and design of the study, acquisition and analysis of data, drafting and critical revision of the manuscript, and approved the final version for publication. Fatma Hande Karpuzoğlu, Ilknur Bingül, Serhat Kılınç, Abdurrahman Fatih Aydın, Aysel Bayram, Semen Önder, Efe Akçe, Semra Doğru‐Abbasoğlu, and M. Uysal conceived and designed the experiments. Fatma Hande Karpuzoğlu, Ilknur Bingül, Efe Akçe, İlayda Sözmen, and Serhat Kılınç performed the experiments. Abdurrahman Fatih Aydın conducted a statistical analysis. Aysel Bayram and Semen Önder performed histopathological evaluations. Fatma Hande Karpuzoğlu, Abdurrahman Fatih Aydın, Ilknur Bingül, Semra Doğru‐Abbasoğlu, and M. Uysal prepared figures and tables. All authors contributed to the drafting and critical revision on the manuscript for important intellectual content. All authors have read and approved the final version of the manuscript.

## Funding

The authors have nothing to report.

## Conflicts of Interest

The authors declare no conflicts of interest.

## Data Availability

The data that support the findings of this study are available from the corresponding author upon reasonable request.
